# Evaluating the Potential for Smoke from Stubble Burning to Taint Grapes and Wine

**DOI:** 10.3390/molecules26247540

**Published:** 2021-12-13

**Authors:** Kerry Wilkinson, Renata Ristic, Imogen McNamara, Beth Loveys, WenWen Jiang, Mark Krstic

**Affiliations:** 1 Department of Wine Science, Waite Research Institute, The University of Adelaide, PMB 1, Glen Osmond, SA 5064, Australia; renata.ristic@adelaide.edu.au (R.R.); imogen.mcnamara@hotmail.com (I.M.); beth.loveys@adelaide.edu.au (B.L.); 2 The Australian Wine Research Institute, P.O. Box 197, Glen Osmond, SA 5064, Australia; maddy.jiang@awri.com.au (W.J.); mark.krstic@awri.com.au (M.K.)

**Keywords:** grapevines, particulate matter, smoke taint, volatile phenols

## Abstract

It has been well established that bushfire/wildfire smoke can taint grapes (and therefore wine), depending on the timing and duration of exposure, but the risk of smoke contamination from stubble burning (a practice employed by some grain growers to prepare farmland for sowing) has not yet been established. This study exposed excised bunches of grapes to smoke from combustion of barley straw and pea stubble windrows to investigate the potential for stubble burning to elicit smoke taint. Increased levels of volatile phenols (i.e., chemical markers of smoke taint) were detected in grapes exposed to barley straw smoke (relative to control grapes), with smoke density and the duration of smoke exposure influencing grape volatile phenols. However, the sensory panel did not perceive wine made from grapes exposed to low-density smoke to be tainted, despite the presence of low levels of syringol providing compositional evidence of smoke exposure. During the pea stubble burn, grapes positioned amongst the burning windrows or on the edge of the pea paddock were exposed to smoke for ~15–20 and 30–45 min, respectively, but this only resulted in 1 µg/kg differences in the cresol and/or syringol concentrations of smoke-affected grapes (and 1 µg/L differences for wine), relative to controls. A small, but significant increase in the intensity of smoke aroma and burnt rubber flavor of wine made from the grapes positioned amongst the burning pea stubble windrows provided the only sensory evidence of any smoke taint. As such, had vineyards been located immediately downwind from the pea stubble burn, it is unlikely that there would have been any smoke contamination of unharvested grapes.

## 1. Introduction

When fires occur near wine regions, smoke can drift into vineyards and, depending on the timing and duration of smoke exposure, can taint grapes, and therefore wine [1,2,3]. Smoke-tainted wines exhibit unpleasant smoky, ashy, burnt rubber and medicinal characters, which have been attributed to the presence of smoke-derived volatile compounds, including phenols such as guaiacol, 4-methylguaiacol, *o*-, *m*- and *p*-cresols, syringol and 4-methylsyringol [4,5,6,7]. Several studies have shown that following grapevine exposure to smoke, volatile phenols accumulate in grapes in glycosylated forms [4,5,8,9,10,11,12,13,14]. As such, both volatile phenols and their glycosides are routinely measured as compositional markers of smoke taint in grapes and wine [15,16,17]. 

Much of the smoke taint research undertaken to date has been in response to recurring incidents of vineyard exposure to bushfire/wildfire smoke and has investigated: the physiological responses of grapevines to smoke exposure [6,18,19]; factors that influence the intensity of smoke taint in wine, including the timing and duration of smoke exposure [1,2], fruit maturity at harvest [20] and grape variety [6]; and strategies that mitigate the impact of smoke taint in the vineyard [12,14,20,21,22,23] or the winery [24,25,26,27]. However, prescribed burning can also result in vineyard exposure to smoke. 

Stubble is the term given to the cut stalks and straw residue remaining in the field after the grains of cereal crops such as wheat, canola, barley, lupins and oats are harvested [28,29,30]. While stubble retention can benefit farming systems through minimization of soil erosion and moisture loss, stubble can negatively affect crop establishment due to blockage of conventional tillage/sowing machinery [28], and therefore yield. As such, stubble burning, i.e., the practice of intentional incineration of stubble, is often used to clear land for reuse [29,30], as well as to minimize carryover of root and foliar diseases and to control pests (especially snails) and weed seed banks [28,30,31]. Research has shown that burning windrows destroys weed seeds more effectively than burning standing stubble, because the higher biomass in windrows increases both the burn temperature and the duration of burning at higher temperatures [31]. Windrows are formed by concentrating the straw residue discarded from the rear of the harvester or by machine raking; burning then occurs several months after harvest, when stubble has cured. 

The smoke and particulates released from stubble burning can have adverse effects on human health and the environment if not properly managed [29,30]. The composition and density of stubble smoke depend on the crop area being burned, the prevailing weather conditions (especially wind speed, which strongly influences smoke dispersion), and the nature of the stubble (i.e., its density and moisture content) [29]. In Australia, policy guidelines and codes of practice have been developed to help land owners and operators manage the off-site impacts of stubble burning (for example [32]) and permits are often required from the local council or fire service to regulate the cumulative effects of regional stubble burning operations.

In regions where grains and wine grapes are grown in close proximity, there is increasing concern that smoke from burning stubble could taint unharvested grapes, especially where burn programs overlap the grape growing season. Although the risk of grapes being contaminated by stubble smoke is likely to be lower than that arising from exposure to bushfire smoke (due to the relative durations of smoke exposure), the potential for stubble burning to taint grapes and wine has not previously been investigated, and was therefore the primary aim of this study. A preliminary trial was first undertaken to confirm that excised grape bunches could be used as ‘sinks’ to monitor smoke exposure during a stubble burn and to establish to what extent smoke density affects temporal changes in grape volatile phenols during smoke exposure, and their carry over into wine. 

## 2. Results and Discussion

### 2.1. Use of Excised Grape Bunches to Monitor Smoke Exposure

#### 2.1.1. Environmental Conditions before, during and after the Preliminary Field Trial

**Figure 1:**
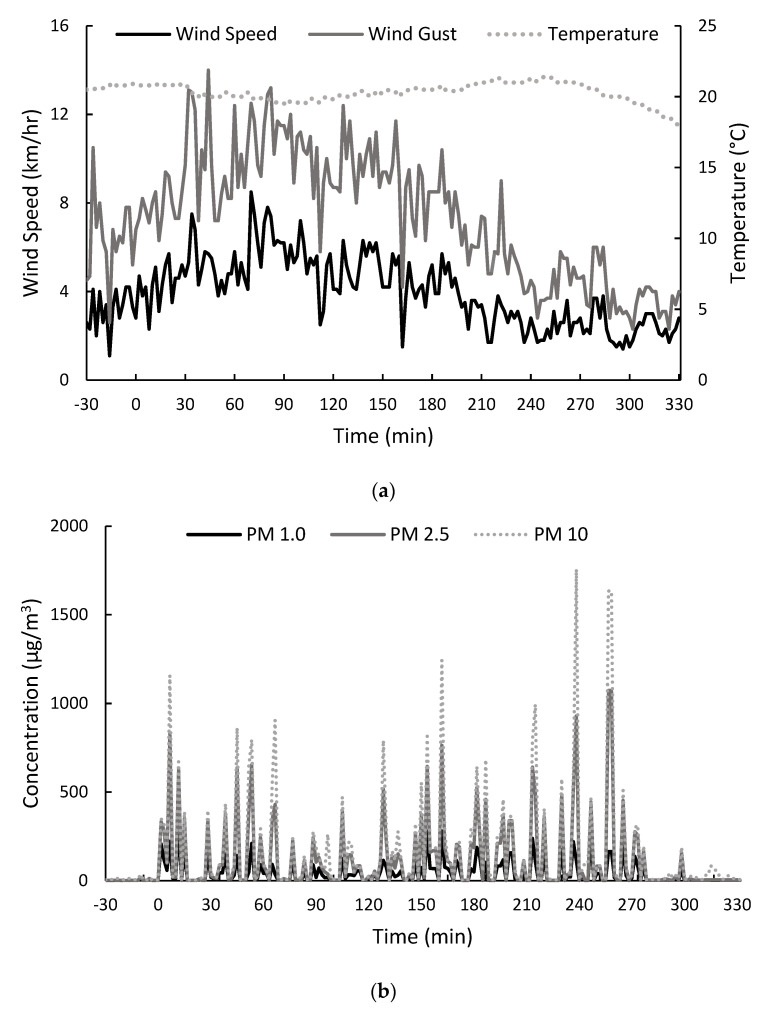
Environmental conditions before, during and after exposure of excised bunches of Shiraz grapes to barley straw smoke (i.e., −30 to 0, 0 to 300 and 300 to 330 min, respectively): (**a**) wind and gust speed, and temperature; and (**b**) particulate matter (PM) concentrations.

Environmental data were collected before, during and after the (5 h) preliminary field trial (Figure 1 and Figure S1). The air temperature remained relatively constant at between 19.5 and 21.4 °C, before dropping to 18.0 °C in the 30 min after the trial (i.e., between 300 and 330 min), whereas wind speeds fluctuated considerably (Figure 1a). Wind and gust speeds increased between 0 and 90 min, peaking at 8.5 and 14.0 km/h, respectively, after which they steadily declined, with only 1.3 to 3.0 km/hr winds and 2.3 to 4.2 km/h gusts observed after 270 min (Figure 1a). Wind direction throughout the trial was west by south-westerly (data not shown). 

Particulate matter (PM) was monitored as a measure of smoke emission (Figure 1b), and as expected, PM fluctuated with changes in wind speed and direction. Similar concentration profiles were observed for the different PM sizes, with PM_10_ > PM_2.5_ > PM_1.0_, in agreement with particle size distributions previously reported for smoke from domestic wood fires [33]. PM was detected immediately after the combustion of barley straw commenced, with lower levels observed when wind speeds were highest and smoke dispersed more quickly (i.e., between 70 and 100 min). With the exception of one time point (i.e., 297 min), PM levels remained below 50 µg/m^3^ after 270 min, which was attributed to the corresponding drop in wind (Figure 1a). Nevertheless, PM data demonstrate the presence and density of smoke throughout the trial.

#### 2.1.2. Density and Duration of Smoke Exposure-Affected Grape Volatile Phenols

**Figure 2:**
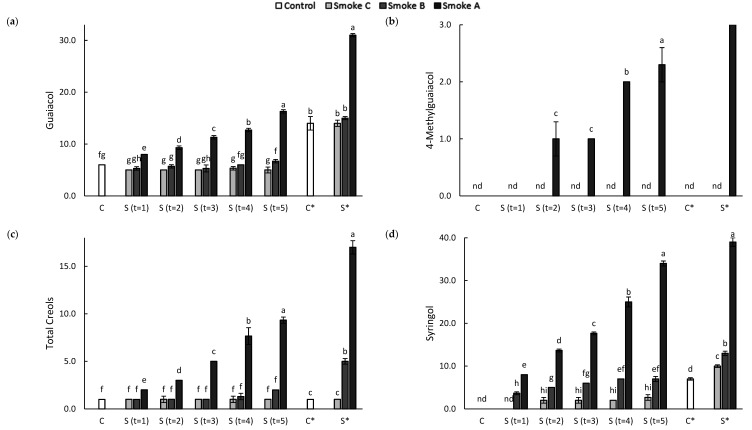
Concentrations of (**a**) guaiacol, (**b**) 4-methylguaiacol, (**c**) cresols and (**d**) syringol in control (C) and smoke-affected (S) Shiraz grapes (µg/kg) sampled at hourly time points (i.e., t = 1, 2, 3, 4 and 5 h) and wines (µg/L, denoted by an asterisk). Data are the means of three replicates ± standard error (where available). Different letters indicate statistical significance (*p* ≤ 0.05, one-way ANOVA) amongst grape and wine samples; nd = not detected.

Only guaiacol and *o*-cresol were detected, at concentrations of 6.0 and 1.0 µg/kg, respectively, as natural constituents of control grapes (Figure 2, Table S1). Smoke-affected grapes from positions B and C, i.e., the positions furthest from the smoke source, were found to have levels of guaiacol and *o*-cresol similar to those of control grapes, irrespective of sampling time. However, syringol was also detected in these samples, at 3.7–7.0 and 2.0–2.7 µg/kg, respectively, after at least 1 h of smoke exposure, with concentrations increasing as a function of the duration of smoke exposure. The presence of syringol in these grape samples provided compositional evidence of their exposure to smoke. Increasing levels of volatile phenols accumulated in grapes at position A, i.e., the position closest to the smoke source, with increasing exposure to smoke throughout the trial. Significantly higher levels of guaiacol, *o*-cresol and syringol were detected in Smoke A grapes, relative to other samples, from as early t = 1; guaiacol and syringol were most abundant, reaching concentrations of 16.3 and 34.0 µg/kg, respectively after 5 h of smoke exposure. *o*-Cresol levels reached 7.3 µg/kg, while *m*-cresol, 4-methylguaiacol and 4-methylsyringol were observed (at up to 3.3 µg/kg), but only after 2 or more hours of smoke exposure. Collectively, these results confirm the accumulation of smoke-derived volatile phenols in grapes depends on both the density of smoke (being a function of the proximity of grapes to the source of smoke) and the duration of smoke exposure. 

#### 2.1.3. Density and Duration of Smoke Exposure Affect Wine Volatile Phenols

The differences in the volatile phenol concentrations observed for grapes were also seen amongst wines (Figure 2, Table S1). Guaiacol, *o*-cresol and syringol were detected in the control wine (at 14.3, 1.0 and 6.7 µg/L, respectively), with similar levels of guaiacol and *o*-cresol being detected in Smoke C wine. However, syringol was present at a significantly higher concentration, i.e., 9.7 µg/L. While the guaiacol content of Smoke B wine was also the same as the control, significantly higher cresol and syringol levels were observed. The highest levels of volatile phenols were found in Smoke A wine, with guaiacol and syringol again the most abundant. Interestingly, whereas the guaiacol concentration of Smoke A wine was almost two-fold higher than the levels detected in grapes (at t = 5), only a 5 µg/L (~15%) difference in syringol was observed between grapes (at t = 5) and wine. The volatile phenol levels observed in this wine were comparable to, or slightly higher than, concentrations reported in several smoke-tainted red wines resulting from field trials involving the application of smoke to grapevines (for 1 h, approximately 1 week after veraison) of different cultivars [6]. 

The volatile phenol glycoconjugate profiles of wines were also determined, but of the glycoconjugates measured, few were detected, and none were found at concentrations above 14 µg/L (Table S2). This was surprising given guaiacol occurs naturally in red grape varieties, including Merlot and Shiraz [34,35], and guaiacol glycoconjugates have previously been found at concentrations of 334 and 1480 µg/L in control and smoke-affected Shiraz wines, respectively [6]. Recent research has shown excised bunches (including table grapes) are capable of glycosylating exogenous volatile phenols [23,36,37], but in the current study, the limited time between smoke exposure and fermentation (~16 h) may not have been sufficient for glycosylation of smoke-derived volatile phenols to occur. Nevertheless, wines were expected to contain higher background levels of volatile phenol glycoconjugates, irrespective of smoke exposure, based on levels reported previously [12,14,15].

#### 2.1.4. Density and Duration of Smoke Exposure Affect Wine Sensory Profiles

Statistically significant differences were observed amongst wines for measurements of residual sugar, pH, malic acid, color and phenolics (Table S3), but could not be attributed to smoke exposure of grapes. Differences were typically small and not considered likely to influence the sensory perception of wines. In contrast, the significant differences observed amongst wine alcohol levels, which varied by as much as 1.1% alcohol by volume (abv), were considered to be capable of impacting wine sensory properties. As a consequence, hotness was included as a palate attribute rated during sensory analysis. However, panelists did not perceive significant differences in the hotness of wines, nor did variation in alcohol content appear to affect aroma and flavor perception (Table S4). 

**Figure 3:**
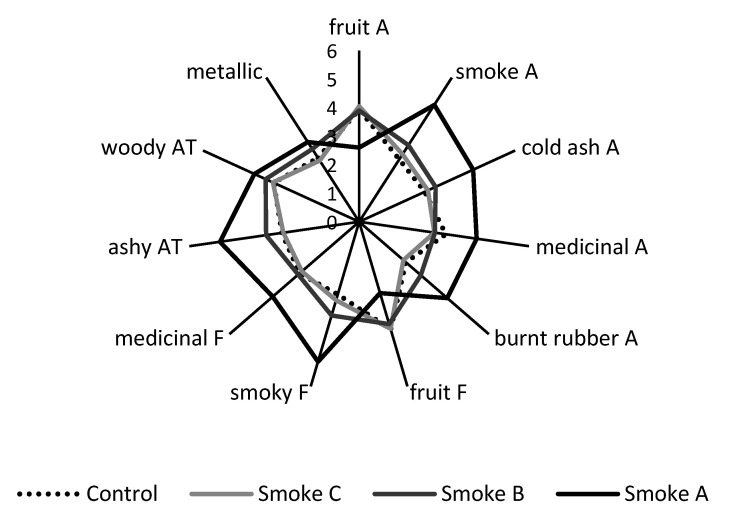
Sensory profiles of control and smoke-affected Shiraz wines; A = aroma, F = flavor, and AT = aftertaste. Data are the mean intensity ratings for one blended wine per treatment, presented to 62 panelists; ratings for all attributes were statistically significant (*p* ≤ 0.05, two-way ANOVA).

The sensory profiles of control and Smoke C wines were very similar, with these wines exhibiting the most intense fruit aromas and flavors, and least intense smoke-related attributes (Figure 3, Table S4). This suggested the panelists did not detect any evidence of smoke taint in Smoke C wine, in agreement with chemical analysis, which found only a small difference in syringol content (Figure 2, Table S1). In the case of Smoke B wines, fruit intensity was comparable with the control, but significantly higher ratings were given for smoke and burnt rubber aromas, and smoky flavor, indicating the panel detected some evidence of smoke taint. In contrast, Smoke A wine was clearly smoke tainted, as evidenced by intense smoky, ashy, medicinal and burnt rubber aromas and flavors, and diminished fruit characters. Again, this was consistent with the compositional data, i.e., significantly elevated volatile phenol levels. No significant differences were perceived amongst mouthfeel attributes, i.e., acidity, hotness, bitterness, or astringency. A study investigating hydrolysis of volatile phenol glycoconjugates by enzymes present in human saliva suggested this phenomenon might contribute to the perception of the ashy aftertaste associated with smoke-tainted wines [38]. However, in the current study, Smoke A wine exhibited the characteristic ashy aftertaste (Figure 3, Table S4), despite volatile phenol glycoconjugates only being detected at exceptionally low levels (Table S2). This suggests both volatile phenols and their glycoconjugates might contribute to the perception of the ashy aftertaste, as reported previously [5]. More importantly, these results demonstrate the use of excised grape bunches as ‘sinks’ for monitoring smoke exposure in research trials (such as the stubble burn trial described in Section 2.2 below) where vineyards are not located in close proximity to the source of smoke to be studied.

### 2.2. Evaluating the Potential for Smoke from Pea Stubble Burning to Taint Grapes and Wine

#### 2.2.1. Environmental Conditions before, during and after the Stubble Burn Trial

**Figure 4:**
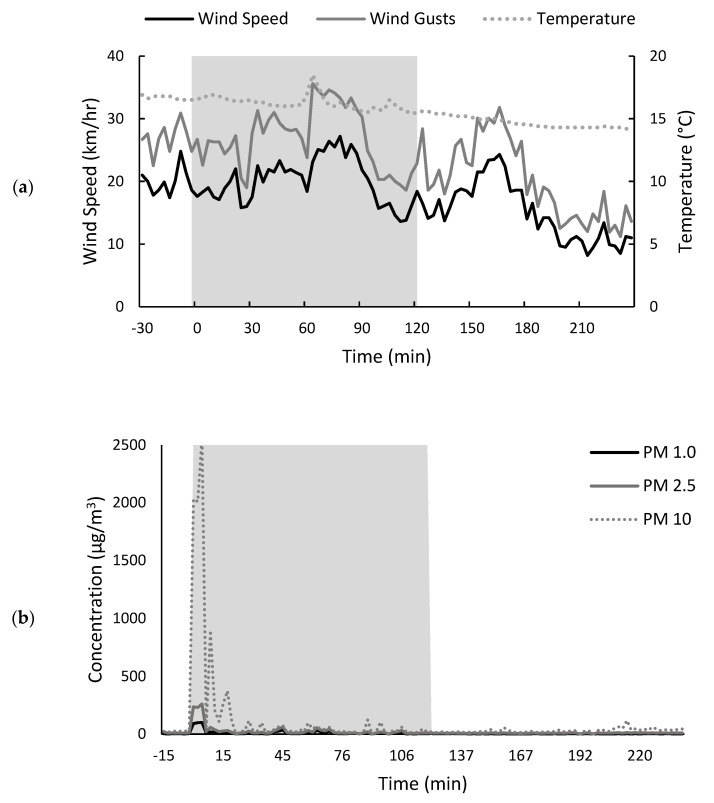

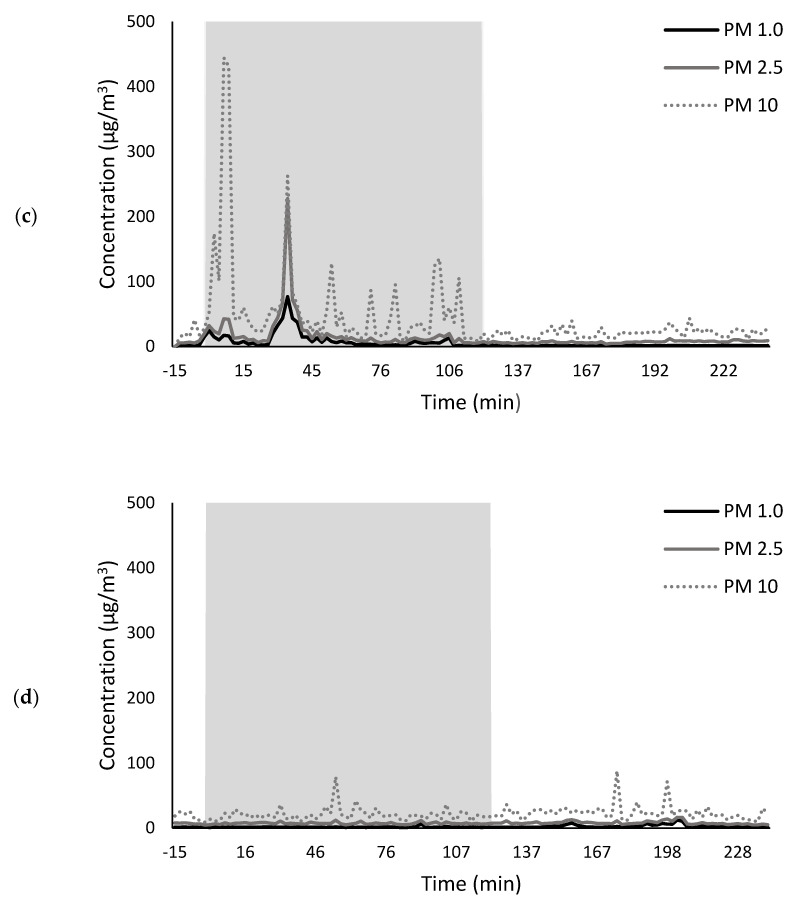
Environmental conditions before, during (shaded) and after pea stubble burn: (**a**) wind and gust speed, and air temperature, measured amongst the stubble windrows; and particulate matter (PM) concentrations, measured (**b**) amongst stubble windrows in a pea field, (**c**) on the edge of the pea field (downwind) and (**d**) in an adjacent field (approximately 500 m downwind).

Environmental data were again collected before, during and after the (~2 h) stubble burn trial (Figure 4 and Figure S2). During this time, the air temperature gradually declined from 16.9 to 14.0 °C, while wind speeds ranged between 13.6 and 27.2 km/h, and averaged 20.1 km/h (Figure 4a), i.e., conditions were slightly cooler and moderately windy, compared to the preliminary trial (Figure 2a). Wind direction throughout the trial was west to south-westerly (Figure S2). 

Prior to the stubble burn, PM levels were <50 µg/m^3^ (Figure 4b–d). The sensor positioned amongst the pea stubble windrows (i.e., sensor A) detected a significant spike in PM levels immediately after the nearby windrows were ignited (Figure 4b), with PM_10_ detected at >2000 µg/m^3^ for ~5 min and then at 50–900 µg/m^3^ for a further ~15 min (Figure 4b). As in the preliminary trial, PM_10_ > PM_2.5_ > PM_1.0_; PM_2.5_ and PM_1.0_ concentrations of ~250 and ~100 µg/m^3^ were observed during the first 5 min after windrows were lit. The sensor position on the edge of the pea paddock (i.e., sensor B, which was ~100 m downwind of sensor A) also detected the smoke plume detected by sensor A, but the smoke had dispersed substantially, such that the PM_10_ concentration peaked at only ~450 µg/m^3^, while PM_2.5_ and PM_1.0_ remained below 50 µg/m^3^ (Figure 4c). This sensor subsequently detected a second smoke plume (at ~35 min into the stubble burn), which originated from combustion of a windrow downwind of sensor A. Throughout the remainder of the stubble burn, sensors A and B detected the occasional presence of smoke, evidenced by recurrent PM_10_ peaks of ~100 µg/m^3^. In contrast, the sensor positioned ~500 m downwind in an adjacent paddock (i.e., sensor C) seemingly did not detect any smoke; with the exception of one time point, PM concentrations remained below 50 µg/m^3^ throughout the stubble burn (Figure 4d). 

The PM data suggest that grapes co-located with sensor A amongst the pea stubble windrows were briefly exposed to dense smoke, then to low–moderate-density smoke over ~20 min, while grapes co-located with sensor B on the edge of the pea paddock were exposed to low–moderate-density smoke for ~30–45 min. The grapes co-located with sensor C, (downwind) in the adjacent paddock, were unlikely to have been exposed to any smoke, due to the combined vertical and lateral dispersion of smoke plumes.

#### 2.2.2. Compositional Consequences of Fruit Exposure to Smoke from Pea Stubble Burn

None of the volatile phenols measured as markers of smoke taint were detected in control grapes and only 1–2 µg/kg of guaiacol and *o*- and *m*-cresols were detected in grapes from the excised bunches deployed during the stubble burn trial, nominally ‘smoke-affected grapes’ (Table 1). Similarly, guaiacol and syringol were each detected in control wine at 2 µg/L, but only 1 µg/L increases in cresols and syringol were observed in wines made from smoke-affected grapes (Table 1). These results suggest grapes were not exposed to sufficient quantities (densities) of smoke, over a long enough period of time, to result in any substantial uptake of smoke-derived volatile phenols, and thus, contamination from stubble smoke. This finding is supported by PM and volatile phenol data from the preliminary trial (Figure 2b, Table S1), which suggest the excised Shiraz bunches positioned closest to the smoke source (i.e., at A and B, Figure S1) were exposed to considerably more smoke (i.e., more dense smoke, for a much longer period of time) than any of the excised Cabernet Sauvignon bunches during the stubble burn trial.

The volatile phenol glycoconjugate profiles of Cabernet Sauvignon wines were also determined (Table S5), but as in the preliminary trial, individual glycoconjugate concentrations were low (<11 µg/L), suggesting the time between smoke exposure and fermentation did not allow for significant glycosylation of smoke-derived volatile phenols. Where small compositional differences were observed amongst volatile phenol glycoconjugate profiles, these could not be attributed to smoke exposure. 

#### 2.2.3. Sensory Consequences of Fruit Exposure to Smoke from Pea Stubble Burn

Statistically significant differences were again observed amongst several of the basic compositional parameters measured in Cabernet Sauvignon wines, being alcohol content, pH, TA, VA, malic acid, wine color and total phenolics (Table S6); but it was not clear to what extent, if any, these could be attributed to smoke exposure of grapes. As in the preliminary trial, most differences in basic chemistry were small and thus, not considered likely to influence the sensory perception of wines. The observed variation in alcohol content (1.4% abv) may have affected the sensory panel’s perception of hotness, but overall, the ratings for this attribute were not significantly different amongst the wines (Table S7), and any apparent differences in wine color were overcome by presenting wines monadically (i.e., one at a time, thereby precluding visual comparisons of samples).

**Figure 5:**
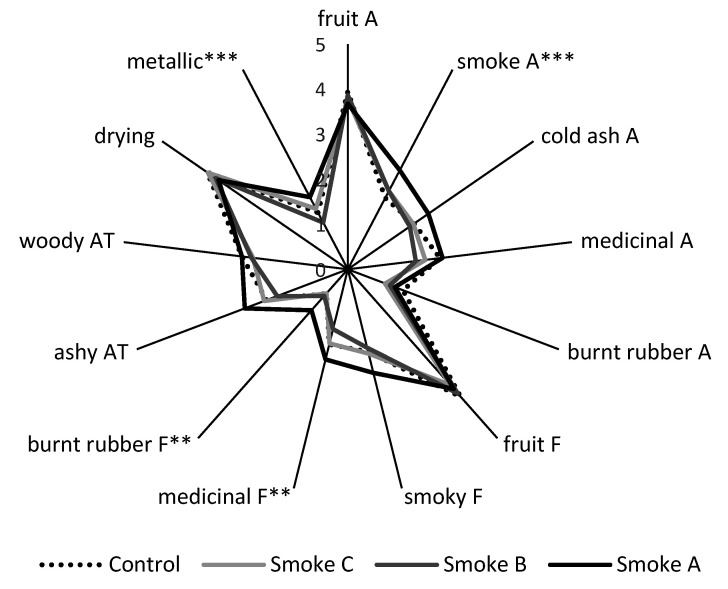
Sensory profiles of control and smoke-affected Cabernet Sauvignon wines; A = aroma, F = flavor, AT = aftertaste. Data are the mean intensity ratings for one blended wine per treatment, presented to 52 panelists; ratings for starred attributes were statistically significant (*** = *p* ≤ 0.05; ** = *p* ≤ 0.01; two-way ANOVA).

The sensory profiles of control and smoke-affected wines were similar (Figure 5, Table S7), with fruit aromas and flavors dominant in all wines, rather than smoke-related attributes. The acidity, astringency, bitterness, hotness and drying mouthfeel character of wines were also perceived to be similar. Only ratings for four attributes were statistically significant. Small, but significant increases in the intensity of smoke aroma and burnt rubber flavor were perceived in the wine made from grapes that were deployed amongst the windrows during the stubble burn (Smoke A wine), relative to other wines (Figure 5), providing evidence of a detectable level of smoke taint. The medicinal and metallic (tinny) flavors of this wine were also rated significantly higher than for the wine made from grapes deployed on the edge of the pea paddock (Smoke B wine), but were not significantly different to other wines (including the control). The sensory data therefore showed good agreement with PM and volatile phenol data, and support the suggestion that exposure of grapes to smoke arising from pea stubble burning only resulted in a perceivable taint where grapes were located amongst the burning windrows. It is worth noting that the perceived level of taint for this wine (i.e., the Smoke A Cabernet Sauvignon wine) was likely lower than that observed for the Smoke B Shiraz wine from the preliminary trial, which displayed smoke and burnt rubber aromas and smoky flavor, and certainly for the Smoke A Shiraz wine, which not only exhibited smoke-related sensory attributes, but diminished fruit aromas and flavors (Figure 3 and Table S4). As such, had a vineyard been located immediately downwind from the pea stubble burn, it is unlikely that there would have been any smoke contamination of unharvested grapes. The prevailing weather conditions (wind speed, in particular) ensured adequate smoke dispersion. The combustion of well-cured pea stubble, pre-raked into windrows, likely aided smoke dispersion as a consequence of improved burn efficiency, i.e., higher burn temperatures are more likely to produce convective heat columns that enhance vertical dispersion of smoke plumes. 

## 3. Materials and Methods

### 3.1. Field Trials

#### 3.1.1. Preliminary Field Trial: Exposure of Shiraz Grapes to Barley Straw Smoke

Shiraz grapes were hand harvested at maturity (i.e., when total soluble solids (TSS) were 27 °Brix) from a commercial vineyard located in the Padthaway wine region of South Australia (36°36′ S, 140°29′ E), and stored in a 0 °C cold room prior to use. The preliminary trial, which involved exposure of grape bunches to smoke over 5 h, was conducted at the University of Adelaide’s Waite Campus. Bunches (60 per treatment) were suspended on wire frames positioned at three distances (2.5, 10 and 35 m) downwind from the smoke source, hereafter positions A, B and C, respectively (Figure S1). Barley straw was combusted in two commercial smokers, with fuel added at 5 min intervals such that a full square bale (weighing approximately 12.5 kg) was burned over 5 h. Grape samples (50 berries in triplicate, per treatment) were collected hourly (i.e., after 1, 2, 3, 4 and 5 h of smoke exposure) and frozen (at −20 °C) for chemical analysis, with berries chosen according to a randomized sampling protocol described previously [39]. A portable environmental sensor (R9 series, Attentis Pty. Ltd., Cheltenham, VIC, Australia) was positioned between A and B (slightly offset from the line of suspended fruit). Environmental data including temperature, relative humidity, wind speed and direction, and the concentration of particulate matter (PM_1.0_, PM_2.5_ and PM_10_) were continuously captured (at ~2 and ~1.6 min intervals for climate and PM measurements, respectively) and uploaded to the manufacturer’s network via an internal Wi-Fi connection. Data were subsequently exported from the network as Excel files. After 5 h of smoke exposure, the remaining fruit was collected and stored overnight (at 0 °C), together with control fruit (i.e., 60 bunches that had not been exposed to smoke), prior to small-scale winemaking. Control fruit was also sampled (50 berries, in triplicate) and frozen (−20 °C) for chemical analysis.

#### 3.1.2. Stubble Burn Trial: Exposure of Cabernet Sauvignon Grapes to Pea Stubble Smoke

Cabernet Sauvignon grapes were hand harvested (when TSS were 24 °Brix) from a commercial vineyard located in the Coonawarra wine region of South Australia (37°17′ S, 140°51′ E), approximately 3 h prior to use. The trial, which involved exposure of grape bunches to smoke from prescribed burning of pea stubble, was conducted in a commercial pea field located in Glenroy (37°14′ S, 140°47′ E), north-west of the Coonawarra. The pea stubble was raked into windrows prior to burning, with the property manager commencing the burn when the prevailing weather conditions (wind, in particular) were in accordance with the local Council’s prescribed burning permit conditions. Bunches (100 per treatment) were suspended on wire frames positioned (i) amongst stubble windrows in the pea field, (ii) on the edge of the pea field (upwind) and (iii) in an adjacent field (approximately 500 m upwind), hereafter positions A, B and C, respectively (Figure S2). Portable environmental sensors (R9 series, Attentis Pty. Ltd.) were positioned alongside the grape bunches at A, B and C. Each sensor continuously captured PM_1.0_, PM_2.5_ and PM_10_ data (at ~1.6 min intervals), while the sensor at position A also recorded temperature, relative humidity, wind speed and direction (at ~2 min intervals), with data again uploaded to the manufacturer’s network via Wi-Fi. On completion of the stubble burn (approximately 2 h after the first windrow was set alight), grape samples (50 berries, in triplicate, per treatment) were collected and frozen (at −20 °C) for chemical analysis. The remaining fruit was collected and stored overnight (at ambient temperature), together with control fruit (i.e., 60 bunches that had not been exposed to smoke), prior to transportation to the University of Adelaide’s Waite Campus for small-scale winemaking. The environmental sensors remained in the field overnight and continued to collect data, which were subsequently exported from the manufacturer’s network as Excel files. 

### 3.2. Winemaking

Grape bunches (~60 and ~100 bunches per treatment for Shiraz and Cabernet Sauvignon, respectively) were destemmed by hand and berries divided (by mass) into three fermentation replicates (per treatment, of ~2.5 kg each). Berries were then crushed by hand, with 50 mg/L additions of sulphur dioxide (added as an 8% solution of potassium metabisulphite) and pH adjustment (to 3.5 with a 10% solution of tartaric acid) made to must prior to inoculation with 300 mg/L Maurivin PDM (AB Biotek, Sydney, NSW, Australia) and addition of 200 mg/L of diammonium phosphate. Musts were fermented on skins at 18–20 °C, with the cap plunged twice daily. Wines were pressed when residual sugar was <1 g/L and cold stabilized at 0 °C for 8 weeks. 

Following cold stabilization, samples (150 mL, per replicate, per treatment) were taken for chemical analysis, before wine replicates were assessed by four sensory experts from the University of Adelaide (all female, aged between 25 and 56 years) to establish there were no off-flavors or obvious differences between replicates. Replicate wines were then blended for sensory analysis (i.e., to give one blended wine per treatment).

### 3.3. Chemical Analysis of Grapes and Wine

Basic wine chemistry parameters were determined by the Australian Wine Research Institute’s (AWRI) Commercial Services Laboratory (Adelaide, SA, Australia) and comprised: alcohol, residual sugar, pH, titratable acidity (TA, as g/L of tartaric acid) and volatile acidity (VA, as g/L of acetic acid) measured using a Foss WineScan analyzer (Mulgrave, VIC, Australia); and red wine color and total phenolics, measured using the modified Somers method and methyl cellulose precipitable tannin assay [40], respectively. The AWRI’s Commercial Services laboratory also measured: (i) volatile phenol concentrations in grapes and wine by GC–MS; and (ii) volatile phenol glycoconjugate concentrations in wine by HPLC–MS/MS; in each case using previously published stable isotope dilution assay methods [9,16,41]. These publications describe the preparation of internal standards, method validation and instrumental operating conditions. Briefly, volatile phenols were measured using an Agilent 6890 gas chromatograph coupled to a 5973 mass selective detector (Agilent Technologies, Forest Hill, VIC, Australia), using *d*_3_-guaiacol, *d*_3_-4-methylguaiacol, *d*_7_-*o*-cresol, and *d*_3_-syringol as internal standards, with a limit of detection of 1–2 µg/L; volatile phenol glycoconjugates were measured using an Agilent 1200 high performance liquid chromatograph equipped with a 1290 binary pump coupled to an AB SCIEX Triple Quad^TM^ 4500 tandem mass spectrometer, with a Turbo V^TM^ ion source (Framingham, MA, USA), using d_3_-syringol gentiobioside as an internal standard, with a limit of detection of 1 µg/L.

### 3.4. Sensory Analysis of Wine

The sensory profiles of wines were determined using the Rate-All-That-Apply (RATA) method [42] with panels comprising 62 participants (23 male and 39 female, aged between 19 and 56 years) for Shiraz wines and 52 participants (22 male and 30 female, aged between 20 and 72 years) for Cabernet Sauvignon wines, with panelists comprising staff and students from the University of Adelaide and the AWRI, as well as regular wine consumers recruited from an in-house database. Prior to wine evaluation, panelists completed a brief induction, during which they were familiarized with both the RATA procedure and a list of attributes and their definitions (Table S8), which were adapted from a previous study [6]. RATA assessments were conducted in sensory booths at 22–23 °C under sodium lights, with wine aliquots (30 mL) presented monadically, in a randomized order, in covered, 3-digit coded 215 mL stemmed International Organization for Standardization wine glasses. Panelists rated the intensity of each sensory attribute using seven-point Likert scales (where 0 = not detected, 1 = ‘extremely low’ and 7 = ‘extremely high’). Panelists rinsed thoroughly with pectin solution (1 g/L) and rested for at least 1 min between samples to mitigate sensory fatigue; water and plain crackers were also provided as palate cleansers. Data were acquired with Red Jade software (Redwood Shores, CA, USA).

### 3.5. Data Analysis

Chemical data were analyzed by one-way analysis of variance (ANOVA) using GenStat (15th Edition, VSN International Limited, Herts, UK). Sensory data were analyzed by two-way ANOVA using participants as a random factor and wines as a fixed factor, with Fischer’s LSD post hoc test (*p* ≤ 0.05), to determine significant differences between wines, using XLSTAT (version 2018.1.1, Addinsoft, New York, NY, USA).

## 4. Conclusions

The preliminary trial demonstrated that smoke from the combustion of barley straw (and presumably therefore, the straw residue found in stubble) can taint grapes, with both the density of smoke and the duration of smoke exposure affecting the uptake of smoke-derived volatile phenols by grapes and the perceived intensity of smoke taint in resulting wines. Interestingly, the sensory panel did not detect smoke taint in a wine made from grapes exposed to low-density smoke, despite the presence of low levels of syringol providing compositional evidence of smoke exposure. The use of excised bunches of grapes as ‘sinks’ for monitoring smoke exposure was also demonstrated and this approach was employed in the subsequent stubble burn trial, along with environmental sensors that measured smoke density and the duration of smoke exposure based on the concentration of particulate matter. Results from the stubble burn trial strongly suggest that the risk of grapes being contaminated by stubble smoke are low, especially when guidelines for managing emissions are followed, e.g., consideration of prevailing weather conditions (wind speed and direction, in particular) and not scheduling burns after rain (when stubble is more likely to smolder than burn), and/or where unharvested vineyards are not immediately downwind from stubble burns. Nevertheless, there are alternative stubble management practices that landowners or operators could consider instead of burning, so as to minimize emissions and their potential impacts on human health and the environment. 

## Figures and Tables

**Table 1:** Concentrations of volatile phenols in control and smoke-affected Cabernet Sauvignon grapes (µg/kg) and wine (µg/L).

Treatment		Guaiacol	4-Methyl Guaiacol	*o*-Cresol	*m*-Cresol	*p*-Cresol	Syringol	4-Methyl Syringol
**Grapes**	Control	nd	nd	nd	nd	nd	nd	nd
	Smoke A	2	nd	1	1	nd	nd	nd
	Smoke B	2	nd	nd	nd	nd	nd	nd
	Smoke C	1	nd	nd	nd	nd	nd	nd
**Wine**	Control	2	nd	nd	nd	nd	2	nd
	Smoke A	2	nd	1	1	1	3	nd
	Smoke B	2	nd	nd	nd	1	3	nd
	Smoke C	2	nd	nd	nd	1	3	nd

Values are means of three replicates (*n* = 3); nd = not detected.

## Data Availability

All data are included in the article and/or Supplementary Materials.

## Supplementary Materials

The following are available online, Figure S1: Schematic of preliminary field trial, indicating relative locations of excised Shiraz grape bunches and the environmental sensor, Figure S2: Schematic of pea stubble burn, indicating relative locations of excised Cabernet Sauvignon grape bunches and environmental sensors, Table S1: Concentrations of volatile phenols in control and smoke-affected Shiraz grapes (µg/kg) and wines (µg/L), Table S2: Concentrations of selected volatile phenol glycosides (µg/L) in control and smoke-affected Shiraz wines, Table S3: Basic composition of control and smoke-affected Shiraz wines, Table S4: Mean intensity ratings for sensory attributes of control and smoke-affected Shiraz wines, Table S5: Concentrations of selected volatile phenol glycosides (µg/L) in control and smoke-affected Cabernet Sauvignon wines, Table S6: Basic composition of control and smoke-affected Cabernet Sauvignon wines, Table S7: Mean intensity ratings for sensory attributes of control and smoke-affected Cabernet Sauvignon wines, and Table S8: Aroma and palate attributes used in sensory analysis.

## Sample Availability

Samples of compounds are not available from the authors.
