# Supplementary material for: Evaluating the Potential for Smoke from Stubble Burning to Taint Grapes and Wine
Source: Molecules. 2021 Dec 13;26(24):7540. doi: 10.3390/molecules26247540 (PMC8703940; doi:10.3390/molecules26247540)
Supplement: Supplementary file 1 [file molecules-26-07540-s001.zip › molecules-1486627-supplementary.pdf]

Evaluating the Potential for Smoke from Stubble Burning to Taint Grapes and Wine

Kerry Wilkinson [1,*], Renata Ristic [1], Imogen McNamara [1], Beth Loveys [1], WenWen Jiang [2] and Mark Krstic [2]

[1] Department of Wine Science, Waite Research Institute, The University of Adelaide, PMB 1, Adelaide, SA 5064, Australia; renata.ristic@adelaide.edu.au (R.R.); imogen.mcnamara@hotmail.com (I.M.); beth.loveys@adelaide.edu.au (B.L.)

[2] The Australian Wine Research Institute, PO Box 197, Adelaide, SA 5064, Australia; maddy.jiang@awri.com.au (W.J.); mark.krstic@awri.com.au (M.K.)

* Correspondence: kerry.wilkinson@adelaide.edu.au (K.W.); Tel: +61-8-8313-7360

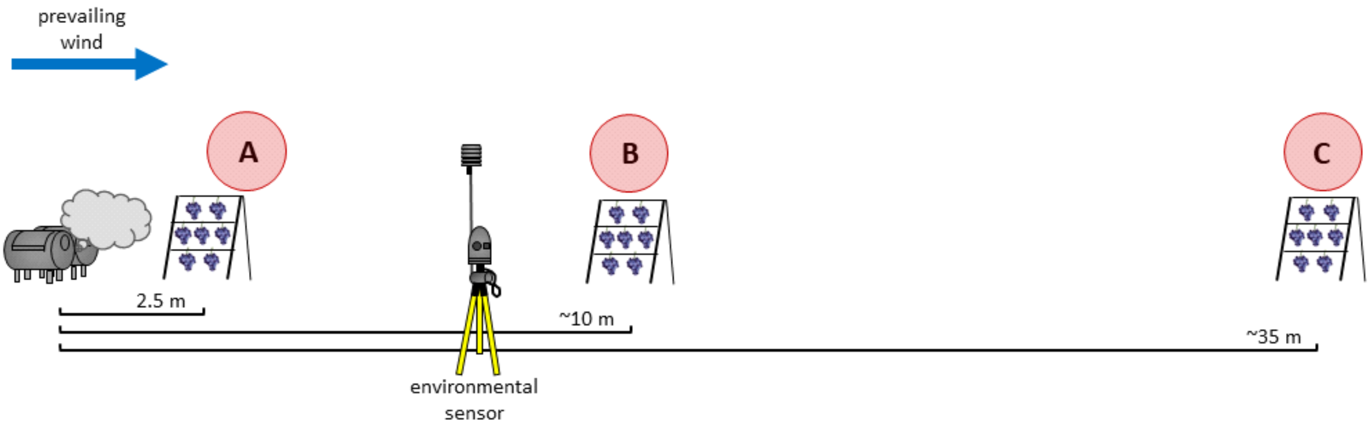

Copyright: © 2021 by the authors. Licensee MDPI, Basel, Switzerland. This article is an open access article distributed under the terms and conditions of the Creative Commons Attribution (CC BY) license (https://creativecommons.org/licenses/by/4.0/).

Figure S1. Schematic of preliminary field trial, indicating relative locations of excised Shiraz grape bunches and the environmental sensor.

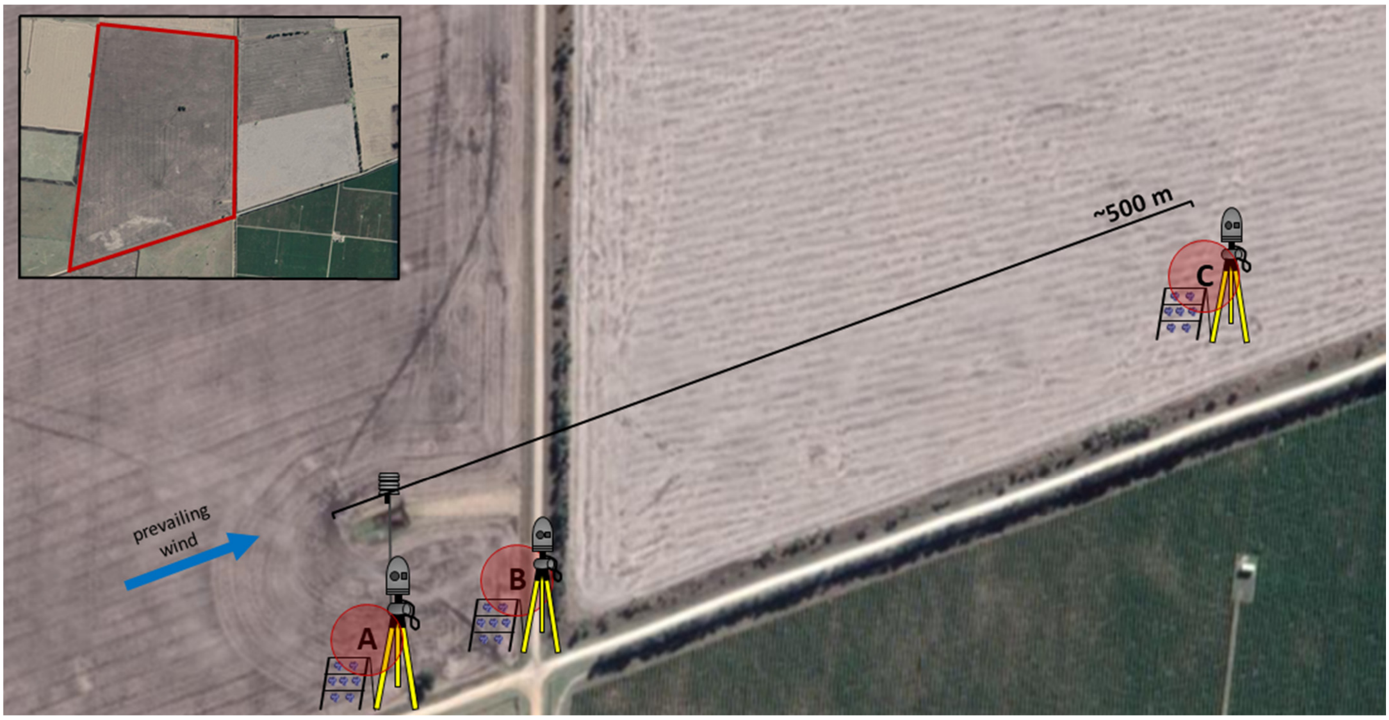

Figure S2. Schematic of pea stubble burn, indicating relative locations of excised Cabernet Sauvignon grape bunches and environmental sensors within the pea paddock (inset); map sourced from *Google Earth*, earth.google.com/web/.

Table S1. Concentrations of volatile phenols in control and smoke-affected Shiraz grapes (μg/kg) and wines (μg/L).

			Guaiacol	4-Methyl Guaiacol	*o*-Cresol	*m*-Cresol	*p*-Cresol	Syringol	4-Methyl Syringol
Grapes	Control		6.0 fg	nd	1.0 f	nd	nd	nd	nd
	Smoke A	t = 1	8.0 e	nd	2.0 e	nd	nd	8.0 e	nd
		t = 2	9.3 d	1.0 c	3.0 d	nd	nd	13.7 d	nd
		t = 3	11.3 c	1.0 c	4.0 c	1.0 c	nd	17.7 c	nd
		t = 4	12.7 b	2.0 b	6.0 b	1.7 b	nd	25.0 b	2.3 b
		t = 5	16.3 a	2.3 a	7.3 a	2.0 a	nd	34.0 a	3.3 a
	Smoke B	t = 1	5.3 gh	nd	1.0 f	nd	nd	3.7 h	nd
		t = 2	5.7 g	nd	1.0 f	nd	nd	5.0 g	nd
		t = 3	5.3 gh	nd	1.0 f	nd	nd	6.0 fg	nd
		t = 4	6.0 fg	nd	1.3 f	nd	nd	7.0 ef	nd
		t = 5	6.7 f	nd	2.0 e	nd	nd	7.0 ef	nd
	Smoke C	t = 1	5.0 g	nd	1.0 f	nd	nd	nd	nd
		t = 2	5.0 g	nd	1.0 f	nd	nd	2.0 hi	nd
		t = 3	5.0 g	nd	1.0 f	nd	nd	2.0 hi	nd
		t = 4	5.3 g	nd	1.0 f	nd	nd	2.0 hi	nd
		t = 5	5.0 g	nd	1.0 f	nd	nd	2.7 hi	nd
	P		<0.001	<0.001	*<0.001*	*<0.001*	–	<0.001	<0.001
Wine	Control		14.3 b	nd	1.0 c	nd	nd	6.7 d	nd
	Smoke A		30.7 a	3.0	6.6 a	9.3 a	2.0 a	39.0 a	4
	Smoke B		15.3 b	nd	2.0 b	2.0 b	1.0 b	12.9 b	nd
	Smoke C		14.0 b	nd	1.0 c	nd	nd	9.7 c	nd
	P		<0.001	–	*<0.001*	*<0.001*	<0.001	<0.001	–

Values are means of three replicates (*n* = 3); nd = not detected. Different letters within columns indicate statistical significance for grapes (P ≤ 0.05, two-way ANOVA; i.e., by treatment and time, where t denotes hours of smoke exposure) and wine (P ≤ 0.05, one-way ANOVA).

Table S2. Concentrations of selected volatile phenol glycosides (μg/L) in of control and smoke-affected Shiraz wines.

	GuR	4MGR	CrR	PhR	SyrGG	4MSGG
Control	11.2 b	2.8 b	4.0 c	1.2 c	nd	nd
Smoke A	13.0 a	3.7 a	6.1 a	1.8 a	2.4	nd
Smoke B	13.5 a	3.9 a	5.0 b	1.6 b	nd	nd
Smoke C	12.7 a	3.6 a	4.9 b	1.5 b	nd	nd
P	0.015	<0.001	<0.015	0.007	–	–

Values are means of three replicates (*n* = 3) measured as syringol glucose-glucoside (gentiobioside) equivalents; nd = not detected. Different letters within columns indicate statistical significance (P ≤ 0.05, one-way ANOVA). Gu = guaiacol; 4MG = 4-methylguaiacol; Cr = cresol; Ph = phenol; Syr = syringol; 4MS = 4-methylsyringol; GG = glucose-glucoside; R = rutinoside.

Table S3. Basic composition of control and smoke-affected Shiraz wines.

	Control	Smoke A	Smoke B	Smoke C	P
alcohol (% abv)	15.0 c	15.2 c	15.6 b	16.1 a	<0.001
residual sugar (g/L)	0.7 a	0.5 a	0.5 a	0.1 b	0.032
pH	3.8 b	3.9 a	3.8 b	3.9 a	<0.001
TA (g/L)	6.67	6.73	7.10	6.67	ns
VA (g/L)	0.47	0.51	0.57	0.39	ns
malic acid (g/L)	1.9 a	1.8 a	1.6 b	1.6 b	0.009
wine color (au)	10.6 b	11.6 ab	11.7 ab	12.6 a	0.019
wine hue	0.53	0.53	0.52	0.55	ns
phenolics (au)	46.7 c	48.7 bc	49.7 b	52.7 a	0.006

Values are means of three replicates ($n = 3$). Different letters within rows indicate statistical significance ($P \leq 0.05$, one-way ANOVA).

Table S4. Mean intensity ratings for sensory attributes of control and smoke-affected Shiraz wines.

	Control	Smoke A	Smoke B	Smoke C	P
fruit aroma	3.94 a	2.60 b	3.90 a	4.05 a	*<0.001*
smoke aroma	2.60 c	4.87 a	3.19 b	2.81 bc	*<0.001*
cold ash aroma	2.53 b	4.39 a	2.94 b	2.65 b	*<0.001*
earthy aroma	3.11	3.50	3.32	3.08	*ns*
medicinal aroma	3.06 b	4.15 a	2.68 b	2.66 b	*<0.001*
burnt rubber aroma	2.16 c	4.08 a	2.86 b	2.05 c	*<0.001*
fruit flavor	3.92 a	2.60 b	3.73 a	3.90 a	*<0.001*
smoky flavor	2.65 c	5.10 a	3.42 b	2.86 c	*<0.001*
medicinal flavor	2.76 b	4.00 a	2.82 b	2.65 b	*<0.001*
ashy aftertaste	2.73 bc	4.92 a	3.29 b	2.68 c	*<0.001*
woody aftertaste	3.29 b	4.03 a	3.60 ab	3.32 b	*0.034*
metallic	2.66 b	3.32 a	3.03 ab	2.55 b	*0.025*
acidity	4.31	4.24	4.29	4.27	*ns*
hotness	3.79	3.87	4.08	4.16	*ns*
bitterness	3.65	3.73	3.48	3.32	*ns*
astringency	4.02	3.84	3.82	3.86	*ns*

Values are means of one blended wine per treatment presented to 62 judges. Different letters within rows indicate statistical significance ($P \leq 0.05$, two-way ANOVA).

Table S5. Concentrations of selected volatile phenol glycosides (µg/L) in control and smoke-affected Cabernet Sauvignon wines.

	GuPG	GuR	4MGR	CrPG	CrR	PhG	PhPG	PhR	SyrPG	SyrGG	4MSGG
Control	7.2 a	1.0	1.8	3.6	1.7	2.6 b	10.8	1.8 a	1.6	1.6	nd
Smoke A	5.5 c	nd	nd	2.7	1.7	3.7 a	9.7	1.4 b	1.4	1.4	nd
Smoke B	6.4 b	nd	1.6	3.2	1.5	2.6 b	10.2	1.3 b	1.4	1.4	nd
Smoke C	7.0 ab	nd	1.5	3.2	1.8	2.5 b	10.5	1.7 a	1.5	1.5	nd

P	0.003	ns	ns	ns	ns	0.011	ns	0.014	ns	ns	–

Values are means of three replicates (n = 3) measured as syringol glucose-glucoside (gentiobioside) equivalents; nd = not detected. Different letters within columns indicate statistical significance (P ≤ 0.05, one-way ANOVA); ns = not significant. Gu = guaiacol; 4MG = 4-methylguaiacol; Cr = cresol; Ph = phenol; Syr = syringol; 4MS = 4-methylsyringol; PG = pentose-glucoside; R = rutinoside; G = glucoside; GG = glucose-glucoside.

Table S6. Basic composition of control and smoke-affected Cabernet Sauvignon wines.

	**Control**	**Smoke A**	**Smoke B**	**Smoke C**	**P**
alcohol (% abv)	14.4 a	13.0 b	14.1 a	14.4 a	0.003
residual sugar (g/L)	0.6	0.4	0.5	0.5	ns
pH	3.4 a	3.3 a	3.4 a	3.4 a	0.049
TA (g/L)	7.33 b	7.87 a	7.47 b	7.50 ab	0.045
VA (g/L)	0.62 a	0.50 b	0.56 b	0.64 a	0.002
malic acid (g/L)	2.2 b	2.8 a	2.2 b	2.2 b	<0.001
wine color (au)	11.2 a	8.6 b	9.7 b	11.4 a	0.003
wine hue	0.52	0.53	0.52	0.50	ns
phenolics (au)	45.0 a	36.3 b	40.3 b	45.7 a	0.002

Values are means of three replicates (n = 3), with the exception of alcohol, residual sugar, pH, TA and VA for Smoke C wine, for which values are means of two replicates (n = 2). Different letters within rows indicate statistical significance (P ≤ 0.05, one-way ANOVA).

Table S7. Mean intensity ratings for sensory attributes of control and smoke-affected Cabernet Sauvignon wines.

	**Control**	**Smoke A**	**Smoke B**	**Smoke C**	**P**
fruit aroma	3.94	3.67	3.87	3.77	*ns*
smoke aroma	1.79 b	2.48 a	1.96 b	1.96 b	*0.034*
cold ash aroma	1.79	2.17	1.67	1.77	*ns*
earthy aroma	2.62	2.92	2.40	2.37	*ns*
medicinal aroma	2.06	2.12	1.52	1.73	*ns*
burnt rubber aroma	1.31	1.12	1.00	0.88	*ns*
fruit flavor	3.77	3.54	3.67	3.42	*ns*
smoky flavor	1.88	2.37	1.79	1.92	*ns*
medicinal flavor	1.71 ab	2.06 a	1.37 b	1.69 ab	*0.008*
burnt rubber flavor	0.71 b	1.21 a	0.79 b	0.71 b	*0.004*
ashy aftertaste	1.92	2.44	1.67	1.98	*ns*
woody aftertaste	2.37	2.37	2.15	2.12	*ns*
drying	3.79	3.48	3.56	3.77	*ns*
metallic	1.42 ab	1.81 a	1.17 b	1.52 ab	*0.034*
acidity	4.33	4.40	4.12	4.25	*ns*
hotness	3.21	3.02	3.13	3.48	*ns*
bitterness	2.98	3.12	2.87	3.10	*ns*
astringency	3.63	3.42	3.42	3.69	*ns*

Values are means of one blended wine per treatment presented to 52 judges. Different letters within rows indicate statistical significance (P ≤ 0.05, two-way ANOVA).

Table S8. Aroma and palate attributes used in sensory analysis.

Attributes	Definition
fruit aroma	Intensity of the overall fruit aroma
smoke aroma	Perception of any type of smoke aroma, including smoked meat/bacon, toasty, charry, cigar-box, estery
cold ash aroma	Burnt aroma associate with ashes, including ashtray, tarry, campfire
earthy aroma	Any aroma associated with musty, dusty, wet-wood, barnyard, mushroom-like, dank, moldy, stagnant, stale
medicinal aroma	Aromatic characteristic of Band-Aids, disinfectant-like, including cleaning products, solvents, chemicals
burnt rubber aroma	Perception of burnt rubber-like aromas
fruit flavor	Intensity of the overall fruit flavor
smoky flavor	Perception of smoke flavor, including bacon and smoked meat
medicinal flavor	Perception of medicinal flavors, including disinfectant-like, cleaning products and solvents
burnt rubber flavor	Perception of burnt rubber flavor
ashy aftertaste	Length of taste associated with residue of ashtray perceived in the mouth after expectorating, including coal ash, ashtray, tarry, acrid, campfire
woody aftertaste	Length of taste associated with woody residue, includes wood, oak, pencil shavings
drying	Intensity of drying, puckering mouthfeel
metallic	The ‘tinny’ flavor associated with metals
acidity	Intensity of sour/acid taste
hotness	Intensity of warmth/heat due to ethanol
bitterness	Intensity of bitter taste, bitter aftertaste
astringency	Intensity of drying, puckering mouthfeel
